# Supplementary material for: Metastasis diagnosis using attenuated total reflection-Fourier transform infra-red (ATR-FTIR) spectroscopy
Source: PLoS One. 2024 May 31;19(5):e0304071. doi: 10.1371/journal.pone.0304071 (PMC11142428; doi:10.1371/journal.pone.0304071)

PCA loading vectors for the colon cancer cells

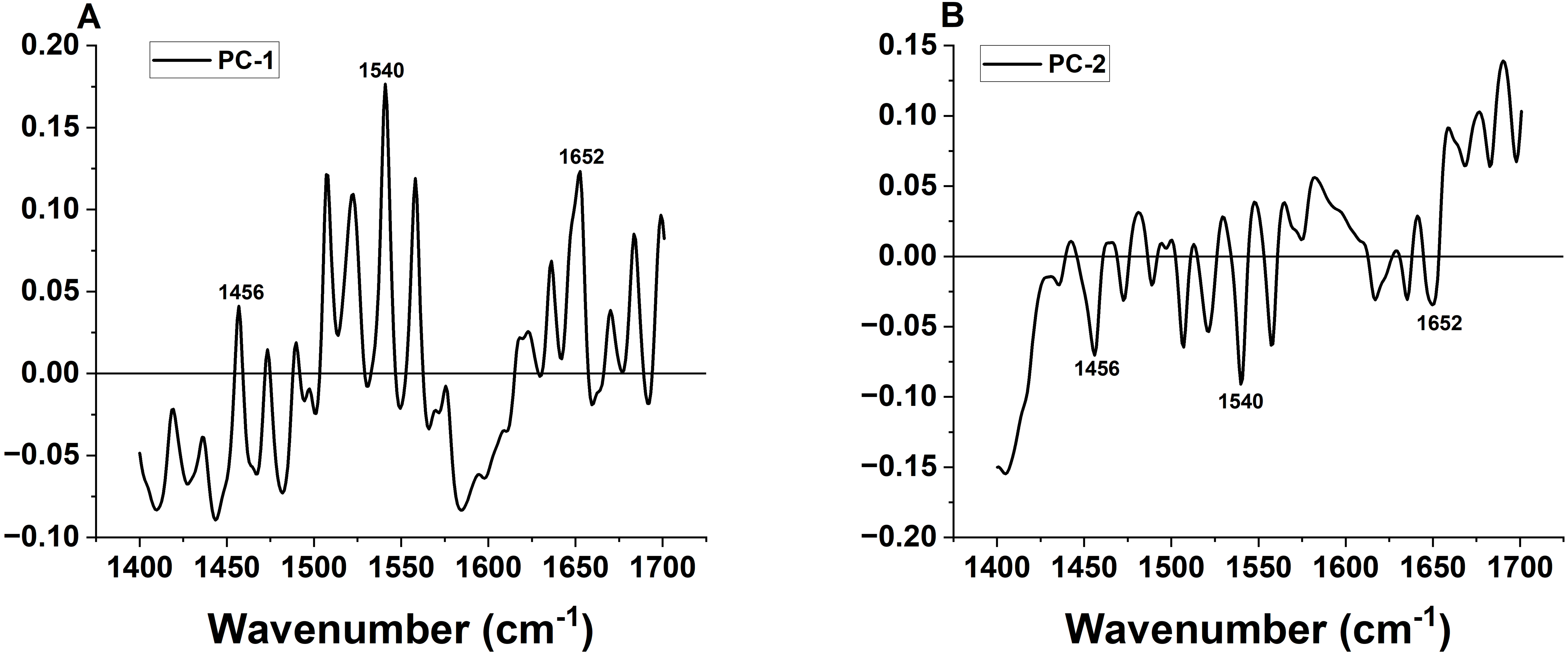

PCA loading vectors for the human melanoma cells

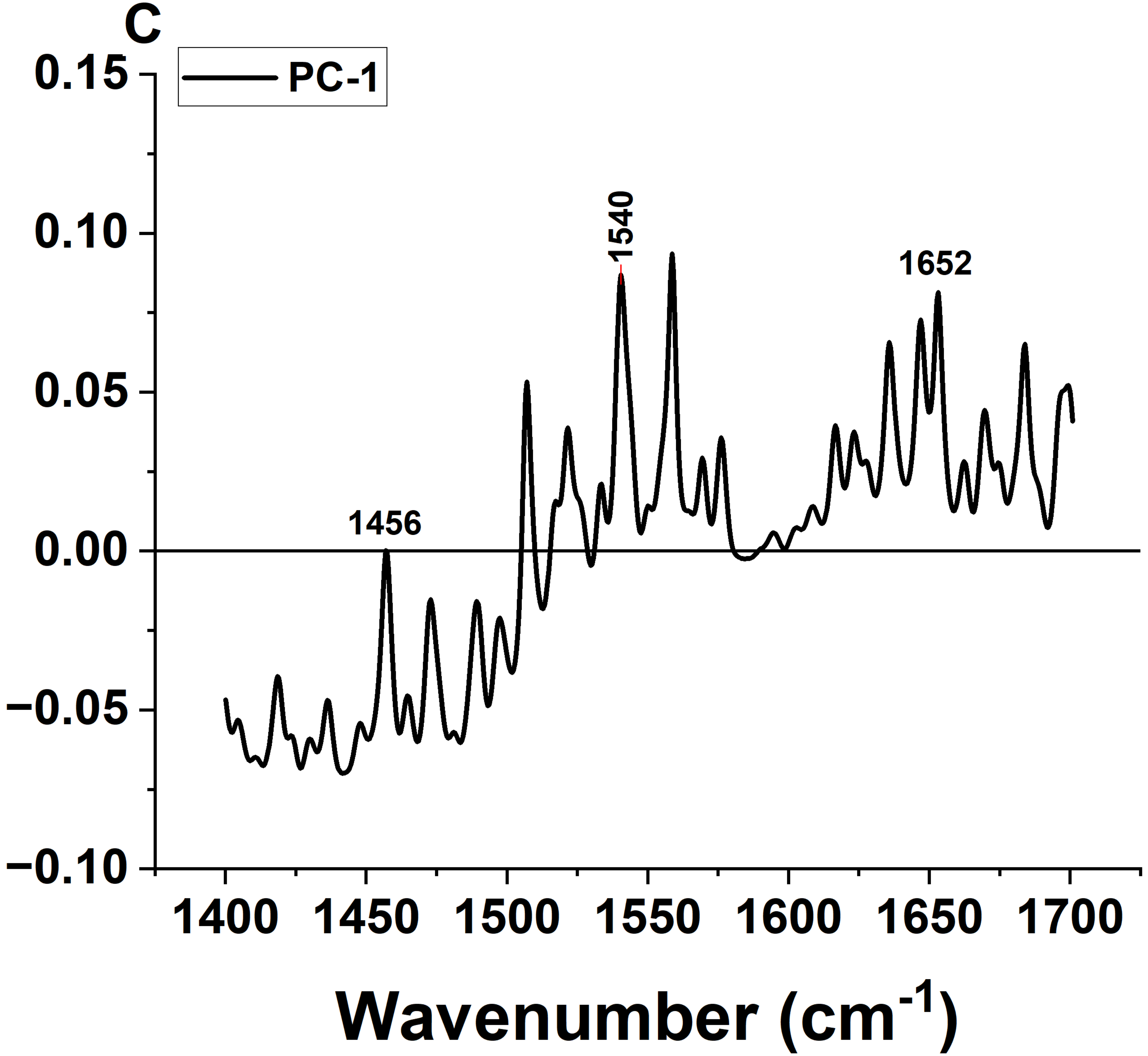

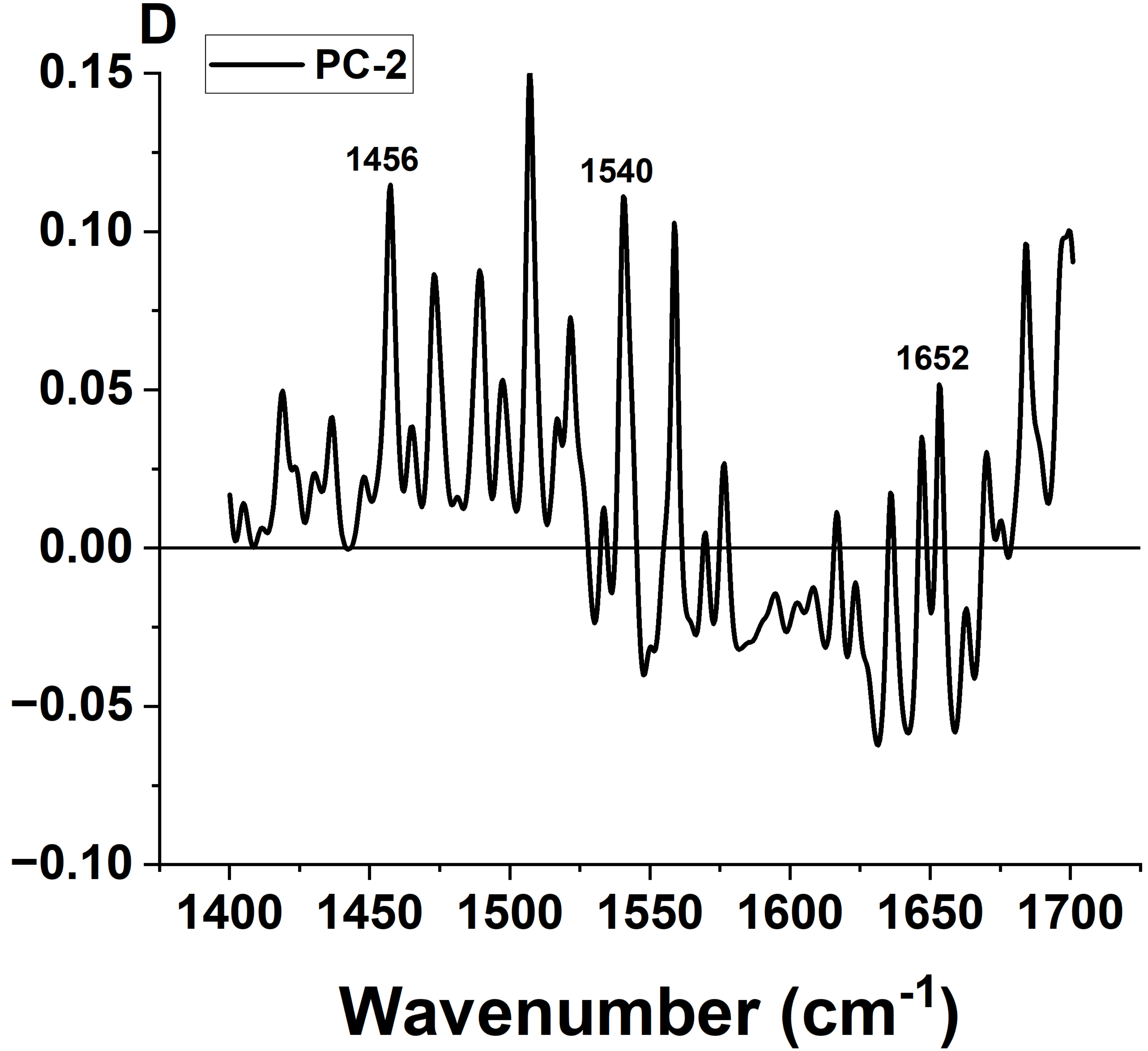

PCA loading vectors for the murine melanoma cells

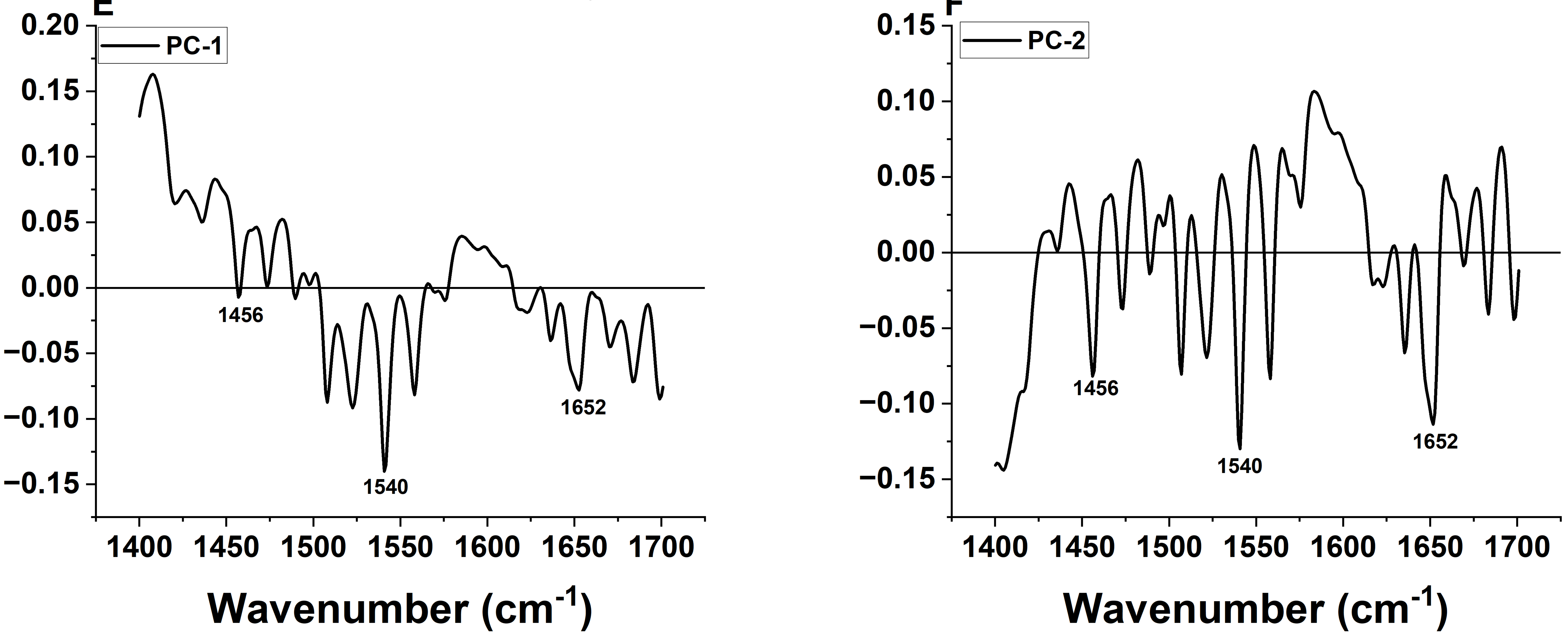

PCA loading vectors for the breast cancer cells

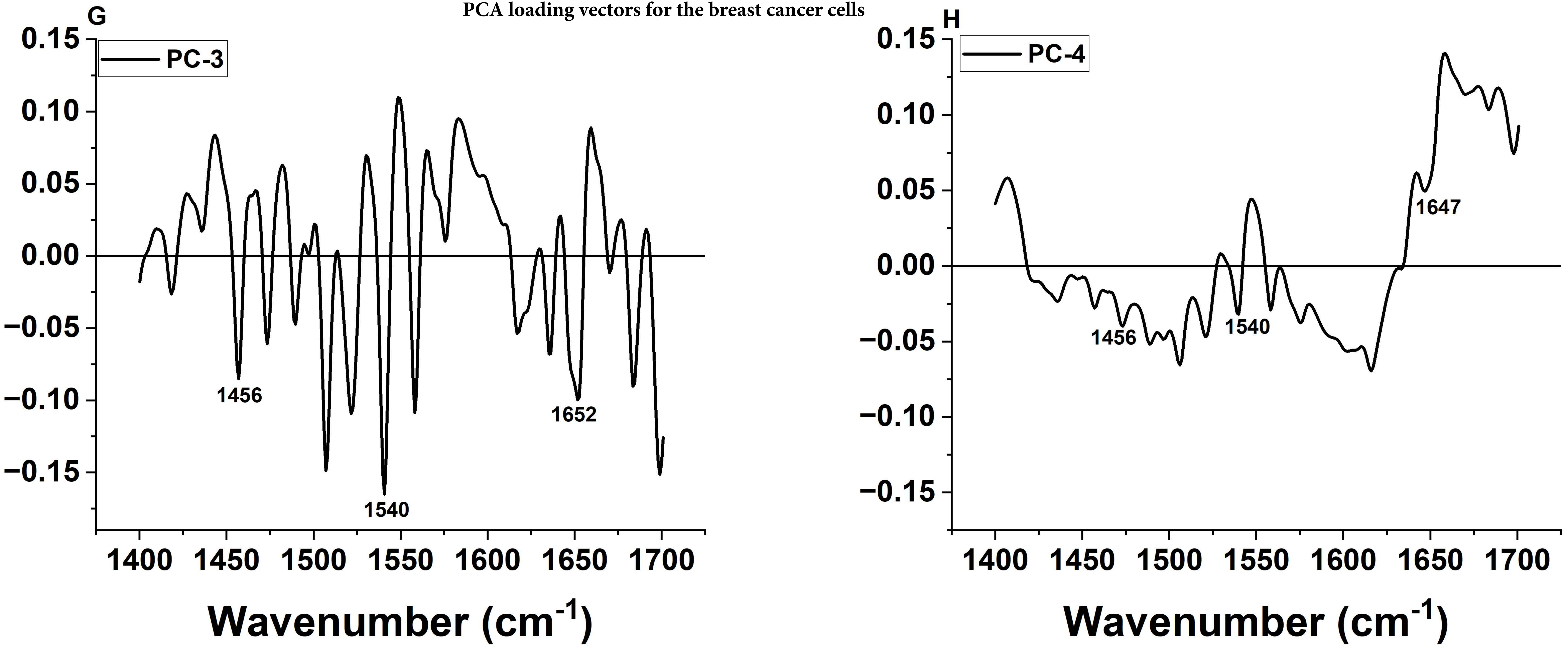

Supplement: S1 File — (A) PC-1 (B) PC-2 loadings for colon cancer cells (C) PC-1 (D) PC-2 loadings for human melanoma cells (E) PC-1 (F) PC-2 loadings for murine melanoma cells (G) PC-3 (H) PC-4 loadings for breast cancer cells. (PDF) [file pone.0304071.s001.pdf]
